# Reintroduction of Legacy Antibiotics in Neonatal Sepsis: The Special Role of Fosfomycin and Colistin

**DOI:** 10.3390/antibiotics13040333

**Published:** 2024-04-05

**Authors:** Maria Baltogianni, Niki Dermitzaki, Chrysoula Kosmeri, Anastasios Serbis, Foteini Balomenou, Vasileios Giapros

**Affiliations:** 1 Neonatal Intensive Care Unit, School of Medicine, University of Ioannina, 451 10 Ioannina, Greece; mbalt@doctors.org.uk (M.B.); nikidermitzaki@yahoo.com (N.D.); f.balomenou@uoi.gr (F.B.); 2 Department of Paediatrics, School of Medicine, University of Ioannina, 451 10 Ioannina, Greece; chrisa.kosmeri@gmail.com (C.K.); aserbis@uoi.gr (A.S.)

**Keywords:** neonatal sepsis, antimicrobial resistance, repurposed antibiotics, colistin, fosfomycin

## Abstract

Neonatal sepsis is a leading cause of morbidity and mortality in neonates, particularly in low- and middle-income countries. The emergence of antimicrobial resistance is a rapidly growing global problem. A significant proportion of the pathogens that commonly cause neonatal sepsis are resistant to multiple antibiotics. Therefore, for the empirical treatment of neonatal sepsis, the repurposing of older antibiotics that are effective against multidrug-resistant pathogens is being investigated. This review aims to provide an overview of current research and experience using the repurposed antibiotics colistin and fosfomycin for the empirical treatment of neonatal sepsis. Based on current knowledge, colistin and fosfomycin may be potentially helpful for the empirical treatment of sepsis in neonates due to their efficacy against a wide range of pathogens and acceptable safety profile.

## 1. Introduction

Neonatal sepsis is a leading cause of neonatal mortality, accounting for more than half a million deaths annually. The highest mortality rates occur in low- and middle-income countries [1,2,3]. The leading causative agents include Gram-negative bacteria such as *Escherichia coli* or *Klebsiella pneumoniae* and Gram-positive cocci such as *Staphylococcus aureus* and *Streptococcus agalactiae* [4]. Current recommendations for first-line treatment of early-onset neonatal sepsis include a narrow-spectrum beta-lactam antibiotic in combination with an aminoglycoside [3,5].

Nevertheless, several studies have shown significant rates of antimicrobial resistance in the above pathogens [6,7,8,9,10,11]. The increased incidence of extended-spectrum beta-lactamases (ESBLs) and aminoglycoside-modifying enzymes is usually the culprit. The two most commonly used antibiotics are a combination of amoxicillin and gentamicin, but resistance rates to these two antibiotics have been reported to be 95% and 60%, respectively [12].

### 1.1. Issues with Prescribing Antibiotics to Neonates

A high percentage of neonatal deaths are attributed to the lack of effective antibiotics currently in use. One issue with antibiotics is the significant delay between testing new antibiotics in adults and their use in neonatal populations. This delay can exceed ten years [13,14]. It has been reported that antibiotics that were approved for use in adults more than 20 years ago have not yet been approved for use in neonates. Additionally, out of the over 40 antibiotics approved for adults in the last 25 years, only four have dosage details for neonatal populations [15]. However, about half of the neonates admitted to neonatal intensive care units (NICUs) receive antibiotics, while about 90% receive antibiotics during their stay [16]. The absence of pharmacodynamic and pharmacokinetic studies in neonatal populations leads to dose extrapolation for older populations while increasing the possibility of inducing antimicrobial resistance once subtherapeutic doses are prescribed. Several studies have documented that microbial resistance is higher in low-income countries, and common multidrug-resistant (MDR) pathogens are as high as 80% in some areas [12,17]. Among the Gram-negative bacteria, *Klebsiella* spp. and *Acinetobacter* spp. play a major role in nosocomial sepsis in the NICU. On the other hand, among the Gram-positive bacteria, *Staphylococcus aureus* and *Streptococcus agalactiae* are the major causes of community-acquired neonatal sepsis [12,18,19].

### 1.2. Definition of Neonatal Sepsis

The definition of neonatal sepsis also creates challenges for effective antibiotic prescribing due to its reliance on microbiological criteria and the fact that it differs from the adult and pediatric definitions [20]. Many symptoms and clinical signs of neonatal sepsis are non-specific and can be observed in other, more benign conditions. Because the definition of neonatal sepsis is variable, the populations included in several clinical trials are also not well defined, and the results of such studies may be questionable [15,21].

Another difference between adults and neonates is that sepsis is diagnosed by a positive blood culture, as neonates rarely present with a focal infection. In adults, however, sepsis is usually the progression of a focal infection [15]. Clinical criteria are often used to diagnose neonatal sepsis rather than relying solely on blood culture results [22]. These differences make it difficult to extrapolate the research results from adult or pediatric populations to neonatal populations.

### 1.3. Older Antibiotics

The need for alternative drug combinations for the initial treatment of neonatal sepsis seems urgent [23]. Two categories of antibiotics are being considered. The first category comprises various antibiotics that have been modified to overcome antimicrobial resistance. The second category includes a few older antibiotics used in the past for different purposes [24]. Many newer antibiotics include a combination of β-lactam + β-lactamase inhibitor, but the microorganisms responsible for neonatal sepsis are resistant to most of these. Under these circumstances, and while some new combinations are in the process of being approved for neonatal use (i.e., cefiderocol, cefepime-taniborbactam, sulbactam + durlobactam), some older antibiotics have been shown to remain active against many pathogens that cause neonatal sepsis [15]. This category of repurposed antibiotics includes two drugs of particular interest to neonatologists: colistin and fosfomycin.

## 2. Materials and Methods

We searched the Pubmed database for relevant studies on using repurposed antibiotics to treat neonatal sepsis up to February 2024. The following keywords were used: “neonatal sepsis”, “neonate”, “sepsis”, “antimicrobial resistance”, “antibiotics resistance”, “Fosfomycin”, and “Colistin”. We also screened the reference lists of the retrieved articles to identify relevant articles that the initial search might not have covered. Ultimately, 70 articles were found, and 41 out of that 70 were included in the present narrative review, specifically randomized control trials, systematic reviews, narrative reviews, and observational studies.

## 3. Colistin

Colistin (polymyxin E) is an antibiotic in the polymyxin group. It is a very old antibiotic, discovered in the 1940s but first used in humans in the late 1950s. At that time, it was used against infections caused by Gram-negative microorganisms, but its use was soon restricted. The development and widespread use of other antibiotics, especially aminoglycosides, regarded as less toxic at the time, gradually limited the use of colistin. The quasi-absence of studies investigating colistin’s efficacy, safety, and pharmacology during the next few decades is remarkable [25].

The increasing problem of the resistance of microorganisms such as *Acinetobacter baumannii*, *Pseudomonas aeruginosa*, and *Klebsiella pneumoniae* has led researchers to reapply this old drug, especially in cases where there are no other treatment options due to multi-resistant strains. Even though colistin is considered the last line therapy for these microorganisms, several cases of colistin resistance have emerged, particularly against *Pseudomonas aeruginosa* [26,27]. Resistance to the other two pathogens, *Acinetobacter baumannii* and *Klebsiella pneumoniae*, is variable [28]. It seems to be increasing, especially for *Acinetobacter baumannii*, whereas resistance to *Klebsiella pneumoniae* is relatively rare. This phenomenon has been observed in several cases [29,30]. A particular problem is hetero-resistance, where the MIC remains unchanged but a subgroup within the strain has reduced susceptibility.

Adaptive and mutational mechanisms are responsible for the development colistin resistance in several microorganisms. Although data on the precise mechanism of resistance are scarce and appear to be bacterial specific, it has been found that two regulatory systems, namely the PmrA-PmrB and PhoP-PhoQ systems, are involved in the development of microbial resistance to colistin [31,32,33]. These systems affect the expression of several bacterial genes because they allow bacteria to react to alterations in the microenvironment. Alterations in the external membrane of Gram-negative bacteria due to mutations render them resistant to the action of colistin. Of note, microbial membranes are the site where colistin acts. Among other factors, environmental pH and magnesium concentrations play a significant role in the expression of several microorganism genes. These mutations ultimately lead to bacterial resistance to colistin [32]. 

The pharmacodynamics and pharmacokinetics of this drug are largely unknown due to its decades of redundancy. New studies based on the current dose of the drug are urgently needed to establish its pharmacodynamics and pharmacokinetics. Similar studies in neonates or children, including a neonatal arm, would be valuable in establishing the best dosing schedules for neonates and infants. Moreover, there is a lack of a universal dose unit, with some products using milligrams and others international units (IU). Even different manufacturers recommend different doses, a situation that can lead to both subtherapeutic and toxic levels of the drug, which in turn can lead to treatment failure and the development of microbial resistance [33].

Limited information exists regarding toxicity. Recent reports have documented that the toxicity of this agent is less than we initially thought [34,35,36,37]. Renal function is not affected after administration of intravenous colistimethate sodium at a dose of 160 mg three times/day. In general, colistin is considered to be less nephrotoxic compared to aminoglycosides [38,39,40]. It has been demonstrated that colistin may affect renal function when combined with aminoglycosides but not when used as monotherapy [39]. It has been shown that colistin can increase serum creatinine levels, but this renal effect is mild and is not usually associated with severe renal effects. Furthermore, it depends on the duration of treatment, whereas a return to normal creatinine levels is observed within one month of discontinuing treatment [41]. In addition to nephrotoxicity, neurotoxicity has also been described. Neurotoxic manifestations such as ataxia, paresthesias, or neuromuscular blockade have been observed, particularly after prolonged treatment, but are usually reversible [42].

### 3.1. Efficacy in Neonatal Sepsis

Several studies have investigated the efficacy of colistin in neonates with multidrug-resistant Gram-negative (MDR GN) sepsis (Table 1) [43,44,45,46,47,48,49,50,51,52,53,54]. 

In a study by Ambreen et al., microbiological clearance was achieved in 82.6% of cases. Factors associated with a microbiological cure included the presence of colistin-sensitive microorganisms and the initiation of colistin treatment within two hours of symptom occurrence. No difference was noted in terms of mortality or microbiologic cure with the administration of additional antibiotics [47]. Cagan et al. also reported no significant difference in microbiological clearance or survival among neonates with culture-proven sepsis, regardless of whether the isolated microorganism was only susceptible to colistin or to the second administered antibiotic as well [53].

Tegkunduz et al. reported a 91.7% efficacy of colistin in neonates with MDR GN-positive cultures. Only a neonate with a ventriculoperitoneal shunt had persistent growth of CSF cultures and was subsequently treated with a combination of intravenous and intrathecal therapy [52]. In another study, 10% (four out of forty) of neonates with culture-proven sepsis had no microbiological clearance of *A. Baumannii*. As two of these had meningitis and ventriculoperitoneal shunts, and two had severe pneumonia and previous abdominal surgery, the authors hypothesized that treatment failure may be due to the presence of a foreign device and limited penetration of the affected area [44]. 

In the aforementioned study, two groups of neonates with sepsis were compared, those who were treated with colistin and those treated with other antibiotics, non-defined by the authors; no significant difference was detected in terms of survival (21.3% vs. 13.6%) [44]. However, in a recent study by Kaya et al., it was reported that neonates receiving colistin had a significantly higher mortality rate compared to the control group (35% vs. 5%). Notably, the neonates in the colistin group were in a more critical condition, as evidenced by the significantly higher percentage of neonates on mechanical ventilation [49].

Ilhan et al. compared the efficacy of colistin in very low birth weight (VLBW) neonates to that in non-VLBW neonates with MDR infection and found no significant difference in favorable outcomes (89.3% vs. 86.8%) [46].

In addition, in a recent review of 17 studies involving 312 neonates with MDR GN infections treated with colistin, Nawkan et al. reported a 94.2% microbiological clearance rate among the patients [55]. 

### 3.2. Safety

The mechanism of colistin-induced nephrotoxicity remains unclear. However, acute tubular necrosis has been proposed as a potential cause. Colistin increases the permeability of the tubular epithelial cell membrane, allowing for the influx of ions and water, ultimately leading to cell lysis. This tubulopathy can result in the electrolyte imbalances that have been observed [36,56]. However, compared with earlier studies, more recent studies have shown a significant reduction in the rate of nephrotoxicity. This is likely due to the use of less nephrotoxic colistimethate sodium, the avoidance of intramuscular administration, increased physician attentiveness to adverse effects, and attempts to reduce concomitant use of other nephrotoxic agents [46,53].

According to the literature, the incidence of nephrotoxicity in adults ranges from 20 to 70% [57]. In a large multicenter study, children aged 13 years or older had approximately seven times the risk of developing nephrotoxicity than younger children. It has been hypothesized that the higher rates of nephrotoxicity reported after childhood may be due to relatively impaired renal function resulting from previous minor insults [58].

Several studies have investigated the risk of nephrotoxicity in neonates treated with Colistin, and the reported rates vary widely (0–26%). Determining the impact of colistin on renal impairment in neonates with sepsis is not straightforward, as neonates with sepsis may present organ dysfunction. Moreover, concomitant administration of other potentially nephrotoxic agents in critically ill neonates is common [45,49]. Co-administration of aminoglycosides and septic shock have been recognized as risk factors for acute kidney injury (AKI) in neonates treated with colistin [43,49]. As nephrogenesis is not completed in neonates born preterm, their ability to handle drugs may be compromised. Additionally, drugs may interfere with the postnatal development of the immature kidney [59]. Evidence suggests that a lower gestational age is associated with an increased risk of nephrotoxicity [43,45,49].

In a retrospective case-control study, Ipek et al. compared neonates who received colistin for proven or suspected sepsis with a control group who received alternative antibiotics, and no significant difference was found in terms of nephrotoxicity (12.8% vs. 13.6%). However, hypokalemia and hypomagnesemia were significantly more common [44]. In contrast, Kaya et al. reported a significantly higher rate of AKI in neonates receiving colistin compared to the control group (26% vs. 1.3%). As the authors hypothesized, this may be partly due to the more critical condition of the neonates in the colistin group, as indicated by higher rates of invasive ventilation and early mortality [49]. Moreover, in a study by Ambreen et al., eight out of one hundred and thirty-five (5.8%) neonates treated with colistin developed AKI and 18.3% developed reversible electrolyte disturbances. The median duration of treatment in this study (8.2 days) was shorter than in the previously mentioned studies [47].

In a study of preterm neonates, the incidence of AKI was found to be 17% [43]. Alan et al. also exclusively studied preterm neonates and reported a 19% incidence of AKI [45]. Electrolyte imbalances, mainly affecting magnesium and potassium, were noted in most neonates in both studies. According to Ilhan et al., the incidence of AKI in VLBW neonates after colistin treatment was not significantly different, although higher, than that in non-VLBW neonates (14.3% vs. 2.6%). However, electrolyte disturbances were significantly more common in the VLBW group [46].

Neurotoxicity is colistin’s second most frequently reported side effect in the literature [49]. The interaction between colistin and high-lipid density neurons and the inhibition of acetylcholine release in the synaptic gap have been suggested as factors contributing to its neurotoxic effects [39]. Of the neurological impacts described, only apnea and seizures are clinically detectable in neonates [44,49]. However, these clinical events in neonates may be attributed to various other factors, such as immaturity, sepsis, or central nervous system comorbidities, and it is not always possible to determine whether they are actual side effects of treatment [46,49,52]. Therefore, although the rate of apneas and convulsions in neonates treated with colistin varies among studies (0–33%), according to the authors, it is impossible to directly attribute them to the treatment due to the presence of comorbidities potentially related to these events in all patients [44,45,46,47,48,49,52,53,54].

### 3.3. Pharmacokinetics

Data for colistin pharmacokinetics in the neonatal population is scarce. Therefore, clinicians use different regimens based on pediatric studies [60]. This is not the ideal approach as neonates have a different physiology to children, leading to expected differences in drug distribution and metabolism. Only one study has investigated the pharmacokinetics of intravenous colistin in neonates by administering a single dose of 150,000 CMS (5 mg[CBA]/mg/kg). According to their report, the maximum concentration occurred at 1.3 ± 0.9 h after administration. In the total population, the concentration of formed colistin 6 h after administration was less than 2 μg/mL, which is the breakpoint for susceptibility to colistin in Gram-negative microorganisms. Moreover, the estimated mean steady-state concentration (C_ave,ss_) was 1.1 ± 0.4 μg/mL [61].

### 3.4. Dosing

Current FDA and EMA recommendations for intravenous colistin administration in children are 75,000–110,000 IU/kg/d (2.5–5 mg[CBA]/kg/d) divided into 2–4 doses. This treatment regimen has been used in most neonatal studies. However, studies in the pediatric population have concluded that this regimen is suboptimal, often resulting in plasma colistin concentrations <2 mg/L [62]. A few studies have evaluated higher doses or the administration or a loading dose, which is routine in adults, in pediatric patients, and reported improved colistin exposure without an increased risk of nephrotoxicity [62,63].

### 3.5. Intraventricular Administration

Studies indicate that the concentration of colistin in the cerebrospinal fluid (CSF) after intravenous administration is suboptimal. This is due to the limited ability of polymyxins to penetrate the blood–brain barrier, which is attributed to their high molecular weight and polycationic structure [64]. Even in the presence of meningeal inflammation, the levels of deposition are insufficient to produce anti-infective activities [65]. A study in neonates showed that the drug concentration in the CSF was significantly higher after intraventricular administration in combination with intravenous administration than after intravenous administration alone [66].

Ambreen et al. reported the outcomes of fifteen neonates with multidrug-resistant GN meningitis treated with colistin. Eight out of the fifteen neonates received intravenous colistin exclusively, and all died. Five neonates received concomitant intravenous and intrathecal treatment and survived. Two neonates received only intrathecal colistin, and one survived [47]. The dose administered was 0.16–0.24 mg/kg, once daily. Among survivors, the median time for CSF sterilization was 3.72 days. The neonate who deceased developed chemical ventriculitis received colistin on alternate days, and CSF sterility was not achieved [64]. This adverse effect has previously been described and observed in up to 11% of patients in a study involving adult and pediatric populations [67].

Intraventricular administration is not approved by the FDA. The European Medicines Agency (EMA) has approved the intraventricular administration of colistin for use in adults. The recommended daily dose is 125,000 IU [68]. Therefore, neonatal dosages are determined empirically based on pharmacokinetic estimates. However, the most commonly used dose in neonates is 0.16–0.24 mg/kg/day, although much higher doses, such as 10 mg/day, have been described [52,64,69].

### 3.6. Inhaled Administration

The penetration of colistin into lung tissue is limited due to its hydrophilic structure; therefore, inhaled colistin has been used in cases of pneumonia to achieve higher concentrations in the lungs. Aerolized colistin is associated with fewer adverse events due to lower systemic absorption compared to the intravenous route [70,71,72,73]. The most common adverse effect reported is bronchoconstriction, which can be managed with b2 agonists or steroids [70,74].

Although it is widely used in patients with cystic fibrosis, several adult studies also report a beneficial effect in cases of MDR ventilator-associated pneumonia (VAP) [71,72]. However, very few studies have evaluated the efficacy and safety of inhaled colistin in neonates [70,72].

Nawkan et al. and Celik et al., in their retrospective studies of eight and two neonates with VAP treated with inhaled colistin, respectively, reported a microbiological cure in all patients with no adverse effects [71,73]. A retrospective case-control study compared the outcomes of neonates with MDR VAP treated with either intravenous colistin or intravenous and aerosolized colistin and concluded that the combination of both forms resulted in better therapeutic outcomes. A significantly higher proportion of neonates in the intravenous plus aerosolized colistin group achieved eradication of the microorganism from tracheal aspirates (68.8% vs. 43.8%), and also significantly lower mortality was noted. As the duration of intravenous administration was shorter in the intravenous plus aerosolized group, significantly less nephrotoxicity was observed compared to the control group [72]. Moreover, the efficacy of inhaled colistin as a monotherapy for the treatment of VAP was evaluated by Kang et al. in a population of eight preterm neonates. Colistin-susceptible *A. baumanni* was isolated from tracheal aspirates in all neonates. Eradication was achieved in the total study population, and no adverse events were observed [74].

The administered inhaled dosage of colistin in neonates varies among studies. Only one study evaluated aerosolized colistin’s pharmacokinetics in six mechanically ventilated neonates with VAP after a single dose of 4 mg/kg [CBA]. The concentration of colistin in the tracheal aspirate remained above the therapeutic threshold of 2 µg/mL for 12 h after administration in 83% of neonates and for 24 h in 50% of neonates. Based on these observations, the authors concluded that a treatment regimen involving the administration of 4 mg/kg twice a day would result in concentrations above the breakpoint in all neonates for the entire 24 h period. It should be noted that the systemic concentration of colistin was low, roughly 30% of the concentration achieved by administration of 5 mg/kg intravenously [75].

## 4. Fosfomycin

Fosfomycin was discovered in 1969 and was derived from the fermentation broth of the *Streptomyces fradiae*. Its original name was Phosphonomycin [76]. Fosfomycin is a natural phosphonic acid with a low molecular weight of 138 g/mol. It has unique molecular characteristics and does not resemble any other antibacterial agent currently in clinical use [77]. Fosfomycin is a bactericidal antibiotic with a distinct mode of action. It inhibits bacterial wall synthesis by binding and inhibiting the cytosolic enzyme MurA, thereby interfering with the construction of the initially formed peptidoglycan chain [77,78,79]. 

It exhibits a wide range of activity against Gram-negative and Gram-positive bacteria, including ESBL-producing and carbapenem-resistant strains [77]. Fosfomycin is capable of biofilm penetration. In addition to eradicating bacteria from biofilms, fosfomycin has been shown to alter the architecture of biofilms in experimental studies [79]. Several pathogens are inherently resistant to fosfomycin, including *Acinetobacter* spp., *Staphylococcus saprophyticus*, *Staphylococcus capitis*, *Chlamydia trachomatis,* and *Mycobacterium tuberculosis* [79,80].

Resistance to fosfomycin can derive from chromosomal mutations in membrane transporters leading to loss of function or reduced number, thereby limiting the intracellular uptake of fosfomycin. In addition, a single amino acid substitution on MurA can diminish the affinity of fosfomycin for MurA, thereby interfering with binding. Moreover, the production of fosfomycin-modifying enzymes can alter fosfomycin’s structure, leading to deactivation [81,82].

Fosfomycin is commercially available as tromethamine salt and as disodium salt for oral and intravenous administration, respectively [78]. The Food and Drug Administration (FDA) has approved oral fosfomycin under the brand name Monural for the treatment of uncomplicated urinary tract infections caused by susceptible Gram-negative organisms. Intravenous fosfomycin sodium, branded IVOZFO™ (Verity Pharmaceuticals, Ontario, Canada), has been approved by the Canadian Food Inspection Agency for adult and pediatric use. Both oral and intravenous preparations are available in many other countries [77,83]. A single 3 g oral dose of fosfomycin tromethamine is available in the US to treat uncomplicated urinary tract infections. This indication is for children 12 years and older and adults [77].

### 4.1. Efficacy in Neonatal Sepsis

The potential usefulness of Fosfomycin has been previously described due to its safety and effectiveness against common MDR pathogens implicated in neonatal sepsis. Furthermore, its mechanism of action minimizes potential cross-resistance to other antibiotic classes [81,84].

A limited number of clinical reports have been published on using fosfomycin in neonates. Taylor et al. described a study of 43 neonates with gastroenterocolitis caused by enteropathogenic *E.coli*. The neonates were treated with high doses of oral fosfomycin (150–200 mg/kg three times daily) for four days. Microbiological cure was achieved in 69.9% of the cases, and clinical symptoms subsided in 88.3% [85]. Furthermore, a few case reports have described favorable outcomes in neonates receiving fosfomycin as a combination therapy, including a case of methicillin-susceptible *Staphylococcus aureus* (MSSA) septicemia with liver abscesses and a case of cerebral abscess caused by *Citrobacter koseri* [86,87].

Although fosfomycin is active against most pathogens involved in neonatal sepsis, it is recommended to use it in combination with other antibiotics due to the rapid development of resistance in vitro and the presence of single-point mutation genes. Moreover, the combined regimen is expected to act synergistically to enhance antimicrobial activity [78]. Darlow et al. conducted a study using static and dynamic in vitro pharmacodynamic models to evaluate the efficacy of combined amikacin and fosfomycin therapy in neonatal sepsis [84]. In a subsequent study, the authors also examined using fosfomycin and flomoxef as an alternative empirical treatment regimen [88]. Both studies demonstrated improved antimicrobial efficacy due to the synergistic effect of the treatment and a reduced risk of resistance compared to monotherapy. The authors concluded that both of these treatment regimens are potentially reasonable options for the empirical treatment of neonatal sepsis, especially in settings with a high rate of resistance [84,88]. 

### 4.2. Safety

Fosfomycin is generally considered safe, with tolerable side effects reported in 1–10% of patients, usually without requiring treatment cessation [89].

The parenteral form of Fosfomycin has a high sodium content of 14.4 mEq/g. The current treatment dosages for neonates involve administering 200–300 mg/kg of intravenous fosfomycin daily, equating to 2.8–4.2 mEq/kg/day of sodium [90]. This poses a potential safety concern in the neonatal population, particularly in preterm neonates, due to their renal immaturity. Furthermore, the ingestion of sodium may result in increased renal excretion as a compensatory mechanism, leading to concomitant potassium loss, potentially causing hypokalemia [78,81]. The oral preparation of fosfomycin has a high fructose content (1600 mg/kg/day), which may increase the risk of adverse gastrointestinal events and affect fluid balance [90].

Based on the literature, Fosfomycin administration is not linked to significant adverse events. A review of 23 studies in the adult population revealed that the most frequently reported adverse effects after intravenous administration were rashes, phlebitis at the site of administration, and hypokalemia. Gastrointestinal disorders were observed following both intravenous and oral administration [91]. Although rare, adult studies have reported cases of heart failure caused by sodium overload [91,92].

However, there is limited evidence regarding its safety in the neonatal population. According to previous case reports, administering up to 200 mg/kg/day of fosfomycin did not result in any adverse effects in neonates [85,86,87,93]. In an open-label randomized controlled trial, Obiero et al. compared the safety of standard-of-care antibiotics (SOC) to SOC antibiotics plus fosfomycin intravenously and orally (SOC-F) in neonates over 34 weeks gestational age with sepsis and no significant difference regarding adverse events was noted (3.2 events/100 infant days, 2.2 events/100 infant days, respectively). It is worth mentioning that neither electrolyte disturbances after intravenous administration nor osmotic diarrhea after oral administration were observed [90].

### 4.3. Pharmacokinetics

The evidence available on the pharmacokinetics of intravenous fosfomycin in the neonatal population is limited (Table 2) [94,95,96,97]. In a previous study, a single intravenous dose of fosfomycin 50 mg/kg was administered to twelve preterm and term neonates with postnatal ages of 1–28 days. The reported Cmax was 96–98 mg/L, and the elimination half-life was reported to be 4.9–7 h. A longer half-life was observed in neonates who received fosfomycin during the first three postnatal days [94]. In the NeoFosfo study, the Cmax reported was 350 mg/L, and the median β phase half-life was 5.2 h after administration of 100 mg/kg [97]. The longer half-life reported in neonates compared to children and adults is likely due to renal function immaturity and the larger distribution volume [78]. 

The aforementioned study examined the pharmacokinetics of orally administered fosfomycin in neonates. The absorption rate and bioavailability after oral administration of 100 mg were reported to be 0.0987/h and 0.48, respectively. The more permeable immature intestinal barrier of neonates possibly explains the much higher values observed in this population compared to adults [97]. In a pharmacokinetic study in a pediatric population, the peak plasma concentration was reported to be up to eight times higher when fosfomycin was administered intravenously compared to oral administration [77]. The mean plasma concentration in the NeoFosfo study was reported to be 70.1 mg/dL after oral administration and 201.7 mg/dL after intravenous administration [77,97].

Fosfomycin is a hydrophilic, low-molecular-weight molecule with negligible plasma protein binding (up to 3%). It is not metabolized but is almost entirely eliminated unchanged through renal excretion [78,79,81]. It can distribute well throughout many tissues, including the kidneys, soft tissues, bones, lungs, muscles, and central nervous system (CNS) [77,79,81,89]. Fosfomycin can penetrate the blood–brain barrier and distribute in the CNS, regardless of the presence or absence of meningeal inflammation [98]. However, higher concentrations in the CNS are achieved in patients with meningitis [78,99]. The CNS distribution is of particular importance in the treatment of neonatal sepsis, as CNS involvement is not uncommon. In the NeoFosfo study, 15 samples of CSF were analyzed, and the CSF/plasma concentrations ratio was estimated to be 0.32 [97]. 

### 4.4. Dosing

The current recommendations from the European Medicines Agency (EMA) regarding fosfomycin dosing regimens in neonates suggest a dosage of 100 mg/kg divided into two doses for neonates with a postmenstrual age of less than 40 weeks and 200 mg/kg divided into three doses for neonates between 40–44 weeks [100]. According to NeoFosfo’s study results, a higher dose of 150 mg/kg twice daily is required to achieve the pharmacodynamic target in neonates and children. For neonates in the first week after birth and VLBW neonates, the recommended treatment is 100 mg/kg administered twice daily [97]. Darlow et al. recently conducted a study using neonatal physiology-based pharmacokinetic (PBPK) models and concluded that a fosfomycin regimen of 100 mg/kg q12h for postnatal days 0–7 and 150 mg/kg q12h for days 8–28 has a high potential of achieving the target levels in both term and preterm infants [101].

In addition to concentration-dependent activity, fosfomycin, as an antibiotic acting by inhibiting cell wall synthesis, also has time-dependent activity. Therefore, many authors propose that regimens with the daily dosage divided into frequent doses, every 6–8 h, may be more efficient, leading to a longer amount of time being spent above the minimum inhibitory concentration (T > MIC) [77,89,102].

## 5. Clinical Points

- Colistin and fosfomycin are potentially useful in treating neonatal sepsis, especially in high-resistance settings.
- These agents are associated with low toxicity, and their use in the neonatal population is considered safe.
- The use of these agents in combination with other antimicrobial agents can minimize the risk of resistance.
- Fosfomycin can penetrate the blood–brain barrier and distribute in the central nervous system, especially in the presence of meningeal inflammation.
- Colistin penetrates poorly in the CNS, and intraventricular administration may be useful in meningitis.

## 6. Conclusions

Investigations into the potential usefulness of legacy antibiotics such as colistin and fosfomycin in the treatment of neonatal sepsis, especially in high-resistance settings, have arisen from the increasing incidence of multidrug-resistant pathogens. Based on current data, these drugs are efficient towards multidrug-resistant pathogens, and their safety profile is acceptable, making them potentially useful alternatives for use in the neonatal population. However, despite the increasing interest in these agents during the last decade, significant gaps remain regarding the optimal administration of fosfomycin and colistin in the neonatal population, including the dosing for both term and preterm neonates and the appropriate combination regimens that reduce the risk of resistance and maximize anti-infective activity.

## Figures and Tables

**Table 1:** Clinical studies reporting on the use of colistin in neonatal sepsis.

	Author	Number of Neonates	Gestational Age, Weeks (mean)	Route of Administration	Dosage	Duration, Days (mean)	AKI *n* (%)	Microbiologic Cure *n* (%)	Survival *n* (%)
1	Aksoy, 2020 [43]	47	27	iv	5 mg/kg/day (q8h)	15.95	8 (17%)	ND	43 (91.5%)
2	Ipek, 2017 [44]	47	32.1	iv iv + ivt: 3 iv + neb: 4	iv: 2.5–5 mg/kg/day (q8h)	18	0 (0)	36 (90%)	33 (70.2%)
3	Alan, 2014 [45]	21	28	iv iv + neb: 1	iv: 2–5 mg/kg/day (q8h)	9	4 (19%)	17 (80.9%)	17 (80.9%)
4	Ilhan, 2018 [46]	66	ND	iv iv + ivt: 1	5 mg/kg/d (q8h)	14	5 (7.5%)	58 (87.9%)	48 (72.2%)
5	Ambreen, 2020 [47]	153	ND	iv iv + ivt: 7 ιv + neb 23 ivt: 2/neb: 3	iv: 2.5–5 mg/kg/day (q6h–q12h) neb: 4 mg/kg/dose twice daily ivt: 0.16–0.24 mg/kg daily	8.2	8 (5.2%)	126 (82.6%)	111 (72.5%)
6	Ambrahams, 2023 [48]	53	29	iv	80,000 IU/kg q12h (<7 days old) 120,000 IU/kg q8h (>7 days old)	5.5	1 (2%)	ND	33 (62%)
7	Kaya, 2024 [49]	77	30	iv	5 mg/kg/d (q8h)	ND	20 (26%)	ND	50 (65%)
8	Al-Lawama, 2016 [50]	21	33	iv	70,000 IU/kg/day	17	0 (0)	19 (91%)	19 (91%)
9	Al-Mouqdad, 2021 [51]	15	27	iv	ND	17	ND	7 (46.7%)	7 (46.7%)
10	Tekgunduz, 2015 [52]	12	31.8	iv iv + ivt: 1	iv: 5 mg/kg/d (q8h) ivt: 10 mg/kg/day	16.9	0 (0)	11 (91.7%)	6 (50%)
11	Cagan, 2017 [53]	65	33.6	iv	5 mg/kg/d (q8h)	15	3 (4.6%)	100%	51 (78.5%)
12	Jajoo, 2011 [54]	18	34.5	iv	50,000–75,000 IU/kg/d (q8h)	13	2 (11.1%)	81%	13 (72%)

AKI: acute kidney injury; iv: intravenous; ivt: intraventricular; neb: nebulized; ND: no data.

**Table 2:** Clinical studies evaluating the pharmacokinetics of fosfomycin in neonates.

Author	Population	Chronological Age (days)	Bodyweight, Mean (gr)	Route of Administration	Dosing	Cmax (mg/L)	Cmean (mg/L)	T1/2 (h)	Conclusions
Molina, 1977 [94]	6, preterm	1–3	1900	iv	50 mg/kg	97.7	ND	7	Higher half-life at earlier postnatal day
Molina, 1977 [94]	5, preterm	21–28	2100	iv	50 mg/kg	96.5	ND	4.9	
Guggenbichler, 1978 [95]	5, term	ND	3400	iv	25 mg/kg	62		2.4	95–98% of the drug was recovered in active form in urine. Slower elimination in neonates than in children.
Guggenbichler, 1978 [95]	5, preterm	ND	1900	iv	25 mg/kg	62	ND	2.8	
Guibert, 1987 [96]	10 term, preterm	ND	ND	iv	200 mg/kg	135	ND	ND	Pharmacokinetic parameters were not altered by different times of infusion (30 min or 2 h).
Kane, 2021 [97]	61	0–3	2800	iv	100 mg/kg	350	201.7	5.2	The oral bioavailability was estimated to be 0.48. Fosfomycin can penetrate CSF following iv and oral administration.
Kane, 2021 [97]	61	0–3	2800	per os	100 mg/kg	ND	70.1	ND	

iv: intravenous, per os: oral administration, ND: no data.
